# Childhood trauma and recent stressors in predicting subclinical psychotic symptoms among Chinese university students in southwest China: a machine learning analysis within a gender-specific framework

**DOI:** 10.1136/bmjment-2025-301761

**Published:** 2025-07-31

**Authors:** Wanjie Tang, Zijian Deng, Zeyuan Sun, Qijun Zhao, Miguel Garcia-Argibay, Kadan Anoop, Tao Hu, Shuang Xue, Natali Bozhilova, Aldo Conti, Steve Lukito, Siqi Wu, Gang Wang, Chunhan Jin, Changjian Qiu, Qiaolan Liu, Jay Pan, Samuele Cortese, Katya Rubia

**Affiliations:** 1 West China School of Public Health and West China Fourth Hospital, Sichuan University, Chengdu, China; 2 School of Computing Science, Sichuan University, Chengdu, China; 3 Department of Child and Adolescent Psychiatry, Institute of Psychiatry, Psychology and Neuroscience, King's College London, London, England, UK; 4 School of Medical Sciences, Örebro universitet, Orebro, Örebro, Sweden; 5 Department of Medical Epidemiology and Biostatistics, Karolinska Institute, Stockholm, Sweden; 6 University of Southampton, Southampton, England, UK; 7 Chengdu Normal University, Chengdu, Sichuan, China; 8 Norwegian University of Science and Technology, Trondheim, Trøndelag, Norway; 9 West China Hospital of Medicine, Chengdu, Sichuan, China; 10 Chengdu University, Chengdu, Sichuan, China; 11 Chongqing Jiaotong University, Chongqing, Chongqing, China; 12 University of Southampton, Southampton, UK; 13 Academic Unit of Psychology and Clinical and Experimental Sciences (CNS and Psychiatry), University of Southampton, Southampton, UK

**Keywords:** Machine Learning, Child & adolescent psychiatry, Data Interpretation, Statistical, Cross-Sectional Studies

## Abstract

**Background:**

Subclinical psychotic symptoms (SPS) are common among college students and can lead to future mental health issues. However, it is still not clear which specific childhood trauma, stressors and health factors lead to SPSs, partly due to confounding factors and multicollinearity.

**Objective:**

To use machine learning to find the main predictors of SPS among university students, with special attention to gender differences.

**Methods:**

A total of 21 208 university students were surveyed regarding SPS and a wide range of stress-related factors, including academic pressure, interpersonal difficulties and abuse. Nine machine learning models were used to predict SPS. We examined the relationship between SPS and individual stressors using χ^2^ tests, multicollinearity analysis and Pearson heatmaps. Feature engineering, t-distributed stochastic neighborhood embedding (t-SNE) and Shapley Additive Explanation values helped identify the most important predictors. We also assessed calibration with calibration curves and Brier scores, and evaluated clinical usefulness with decision curve analysis (DCA) to provide a thorough assessment of the models. In addition, we validated this model using independent external data.

**Findings:**

The Extreme Gradient Boosting (XGBoost) model had the best prediction results, with an Area Under the Curve (AUC) of 0.89, and validated with external data. It also showed good calibration, and DCA indicated clear clinical benefit. Interpersonal difficulties, academic pressure and emotional abuse emerged as the strongest predictors of SPS. Gender-stratified analyses revealed that academic pressure and emotional abuse affected males more, while health issues like chest pain and menstrual pain were stronger predictors for females.

**Conclusions:**

Machine learning models effectively identified key stressors associated with SPS in university students. These findings highlight the importance of gender-sensitive approaches for the early detection and prevention of psychotic symptoms.

**Clinical implications:**

SPSs in college students can be predicted by interpersonal difficulties, academic stress and childhood emotional abuse. This information can help mental health professionals develop better ways to prevent and address SPSs.

**WHAT IS ALREADY KNOWN ON THIS TOPIC**

- The close associations between childhood trauma, stressful life events, health factors and subclinical psychotic symptoms (SPS) have already been established. However, it remains unclear which specific types of trauma and stressful events have the greatest impact. Comprehensive predictive models based on these factors have not yet been developed, especially for undergraduate populations.

**WHAT THIS STUDY ADDS**

- By applying nine machine learning models, this study identified the optimal predictive model and found that interpersonal difficulties, academic stress and childhood emotional abuse are the three most influential factors for SPS. Additionally, gender differences were observed: males face higher risk mainly due to greater academic stress, while pain symptoms have a stronger impact on females. The model’s performance was also validated using external data.

**HOW THIS STUDY MIGHT AFFECT RESEARCH, PRACTICE OR POLICY**

- This study provides a foundation for further optimisation and refinement of predictive models for assessing SPS risk in college students, as well as the development of targeted intervention strategies. It also offers clinical guidance for prevention and early intervention.

## Introduction

 Subclinical psychotic symptoms (SPS), such as thought control, paranoia and strange experiences, are prodromal symptoms of psychotic disorders.^1^ It is reported that the prevalence of SPS among the general population ranges from 7.2% to 26.69%.^2^ Among individuals with SPS, 20% reported persistent symptoms, and about 7% eventually developed a psychotic disorder,^3^ which was associated with adverse outcomes. Additionally, SPS was associated with an increased risk of a variety of mental disorders beyond psychotic disorders, including affective, externalising and substance use disorders as well as poorer social function.^4^

Despite the existence of numerous aetiological models predicting SPS, they are sometimes constrained by limited sample sizes or an imbalance between clinical relevance and statistical significance. A growing body of evidence has suggested some potential risk factors such as childhood trauma, stressful life events, somatic symptoms and socioeconomic status, including pain symptoms and low family income.^5^ However, previous studies have neglected potential confounding effects and multicollinearity by considering risk factors in isolation, leading to possible misleading statistical results.^6^ To address this, a large-scale, data-driven study is needed to evaluate the combined impact of these factors, enabling more accurate predictions of SPS by considering their confounding effects rather than examining them independently.

Childhood trauma, including emotional, physical and sexual abuse, as well as neglect, is a well-established risk factor for SPS. Research has shown that exposure to trauma increases the likelihood of developing SPS.^5 7^ However, studies have shown mixed results about how different types of maltreatment affect SPS. For example, Rössler *et al*^8^ found links between SPS and physical or emotional abuse and neglect, but not sexual abuse.^8^ In contrast, Fekih-Romdhane *et al*^9^ found a link only with sexual abuse.^9^ Additionally, many existing studies have small sample sizes and other research limitations. This means we need larger, better-designed studies to better understand the link between childhood trauma and SPS.

According to the stress-vulnerability model,^10^ stress can trigger psychiatric symptoms when it exceeds an individual’s coping capacity. For instance, a college study found significant associations between stressful life events and SPS,^11^ while another study reported that adolescents with higher daily stress exhibited more prodromal psychotic symptoms.^12^ However, some studies have found no link between life events and psychotic symptoms in young adults,^13^ highlighting the need for further investigation into the relationship between stressful life events and SPS.

Left-behind children (LBC) are also a potential risk factor affecting SPS. China once had tens of millions of LBC which refers to those in poor rural areas whose young parents leave home to work in cities, leaving their young children in the care of grandparents or other relatives.^14^ Furthermore, since Chinese culture often favours boys, stress may affect boys and girls in different ways. Therefore, this study will examine LBC and gender differences to better understand these issues. By identifying which childhood problems and stressful events have the greatest impact on SPS, we can offer better support to students in China.

Modern machine learning (ML) techniques have enabled researchers to examine multiple lines of risk factors simultaneously with increasing analytic rigour. ML techniques are superior to traditional statistical methods such as logistic regression in identifying risk factors in epidemiological studies in high dimensional data when there is poor a priori knowledge of the potential associations. Therefore, this study employed ML techniques on a comprehensive dataset of Chinese undergraduate students to identify potential risk factors for SPSs. The analysis took into account variables such as childhood trauma, recent stressful life events and relevant sociodemographic and pain-related data.

## Methods

### Participants and procedure

Participants were undergraduate students from three universities in Southwest China. Because China’s enrolment policy assigns quotas to each province and ethnic group, the student body is nationally representative and diverse. Therefore, our sample includes students from many regions and ethnic backgrounds across the country.

Twenty research assistants and 150 assistant teachers were trained for 2 hours to help with the online survey. Participants could ask questions through WeChat or email and got quick answers. The survey link and informed consent form were shared in class WeChat groups. Participation was voluntary and students could withdraw at any time. After completing the survey, they received a small reward (1–3 RMB). To check data quality, we included simple attention questions, like ‘What is the capital of China?’ Surveys finished in less than 10 min were excluded.

A total of 31 602 students were invited to join the survey through WeChat groups. The online survey, run on the Wenjuanxing platform between March and April 2022, was completed by 21 534 students, giving a response rate of 68.1%. After removing 326 students who failed validity checks, the final sample included 21 208 students, with an average age of 19.71 years (range 16–30). To check for selection bias, we compared basic information (age, gender, grade and major) between participants and non-participants and found no significant differences (online supplemental table 1). This suggests selection bias was minimal.

### Measures

#### Subclinical psychotic symptoms

SPS were measured over the past month using the *psychoticism* and *paranoid ideation* subscales of Symptom Checklist-90-R (SCL-90-R) Chinese version.^15^ Participants responded on a five-point Likert scale (0 = ‘*not at all*’, 1 = ‘*a little bit*’, 2 = ‘*moderately*’, 3 = ‘*quite a bit*’ and 4 = ‘*extremely often*’). Categories of distress for both subscales were defined as follows: ‘*no distress’* was indicated by a mean value of less than 1.00, while a mean value greater than 1.00 was classified as ‘*positive*’. This classification follows the criteria used in previous studies.^1^ Example items included: ‘Someone else can control your thoughts’, and ‘Hearing voices others do not hear’. In the present study, Cronbach’s α was 0.905 for the full SCL-16 SPS scale, 0.804 for the paranoid ideation subscale and 0.849 for the psychoticism subscale.

#### Stressful life events

Stressful life events were assessed using the modified Chinese Adolescent Self-Rating Life Events Checklist,^16^ a widely used tool with demonstrated reliability and validity for measuring stressful events in young adults. The 26-item scale covers five areas: interpersonal difficulties, academic pressure, punishment, personal loss and health/adaptability. In the current sample, Cronbach’s α for the total scale was 0.892. Cronbach’s α for the subscales also indicated moderate reliability: interpersonal difficulties, 0.778; academic pressure, 0.746; being punished, 0.679; personal loss, 0.693; and health and adaptability, 0.637.

#### Childhood trauma

Childhood trauma was assessed using the 28-item Childhood Trauma Questionnaire (CTQ),^17^ which measures five types of trauma experienced before age 16: emotional abuse, physical abuse, sexual abuse and emotional and physical neglect. Participants were asked to rate the childhood trauma on a 5-point scale (from 1=*never* to 5=*always*). In this study, the full scale had excellent reliability (Cronbach’s α=0.864), with satisfactory values for emotional abuse (0.765), physical abuse (0.762), sexual abuse (0.783), emotional neglect (0.942) and physical neglect (0.659).

#### Depressive symptoms

The Patient Health Questionnaire-9 (PHQ-9) is a widely used instrument designed to screen for the severity of depression. It has been validated in a Chinese population.^18^ The PHQ-9 is a nine-question tool used to check how often someone has experienced depressive symptoms in the past 2 weeks. Each question is scored from 0 to 3, with 0 meaning ‘not at all’ and 3 meaning ‘nearly every day’. Total scores range from 0 to 27. Higher scores show worse depressive symptoms. In this study, the PHQ-9 demonstrated excellent reliability (Cronbach’s α=0.854).

#### Sociodemographic and health-related information

We gathered a range of factors based on previous research, including family history of mental illness, romantic relationships and LBC status. In addition, we collected demographic information such as age, gender, year of study and major. Health-related data were also collected, including information on chronic headaches and pain in the back, neck, chest and joints. Participants self-reported if they had frequently experienced any of these types of pain in the past year to an extent that interfered with their work or studies.

### Statistical analyses

All statistical analyses were performed using SPSS V.22.0 (IBM, Chicago, Illinois, USA) for descriptive statistics, and Python V.3.9.17 with sklearn V.1.3.0 for ML methods. Descriptive statistics were used to summarise sociodemographic and clinical information. χ^2^ tests checked for differences in SPSs across sociodemographic groups, childhood abuse types and stressful life events. We calculated the Variance Inflation Factor (VIF) to check for multicollinearity among the independent variables. A Pearson correlation heatmap was also created to visually show how the independent variables are related to each other.

The nine ML models were chosen based on experience from previous model selection in the literature^19^: Gradient Boosting Tree, Logistic Regression, Random Forest, Support Vector Machine, Multilayer Perceptron, the Extreme Gradient Boosting (XGBoost), Naive Bayes, k-nearest neighbours, CatBoost, AdaBoost (see online supplemental section 1 for details). By evaluating these models, we aim to identify the optimal methodological approach. Participants were randomly divided into training (80%) and testing (20%) sets for each fold. Model performance was evaluated across folds using metrics such as average AUC, balanced accuracy and Area Under the Precision-Recall Curve.

The performance of the optimal ML model was evaluated using Receiver Operating Characteristic (ROC) and P–R curves, along with metrics such as sensitivity (Sen), specificity (Spe), positive predictive value (PPV) and negative predictive value (NPV). We also performed a decision curve analysis for the XGBoost model to assess clinical utility, and calculated the calibration curve and Brier score to evaluate the model’s calibration performance. To further explore the impact of individual variables on SPS, we applied Shapley Additive Explanation (SHAP) to quantify the contribution of each feature to the model’s predictions. SHAP waterfall plots were used to provide detailed insights into how individual features influenced predictions for specific samples. Additionally, feature engineering and t-SNE visualisation were employed to effectively display how features distinguished positive and negative samples in 2D space.

A key aspect of our analysis was evaluating algorithmic fairness and potential gender bias. To further investigate the relationship between sexual abuse and SPS, we used a mediation model to examine how depression mediates this relationship.

To validate the performance of our prediction model, we will employ an independent, non-public dataset comprising approximately 8000 university students recruited from universities in southern and southwestern China.

## Results

### Clinical features, multicollinearity assessment and χ^2^ test results

Approximately 15% of participants reported experiencing SPS, with 7.4% endorsing paranoid ideation (mean (SD): 2.42 (3.68)) and 13.8% endorsing psychoticism (mean (SD): 1.65 (2.47)).

The heatmap confirmed generally low inter-variable correlations (online supplemental figure 1), with only a few pairs showing moderate to high correlation coefficients (|r|>0.8). Furthermore, the multicollinearity assessment showed that most variables had VIF values below 2 (online supplemental figure 2), with the highest being physical abuse (VIF=4.25), which is still well below the commonly accepted thresholds (VIF >5 or 10). Overall, these results show that multicollinearity is not a major concern in our study.

χ^2^ tests showed that students with a history of psychological or sexual abuse were more likely to have SPS (see online supplemental table 2). SPS were also more common in students with high academic pressure and interpersonal difficulties. We used False Discovery Rate correction to adjust p values for multiple comparisons.

### ML results

**Figure 1:**
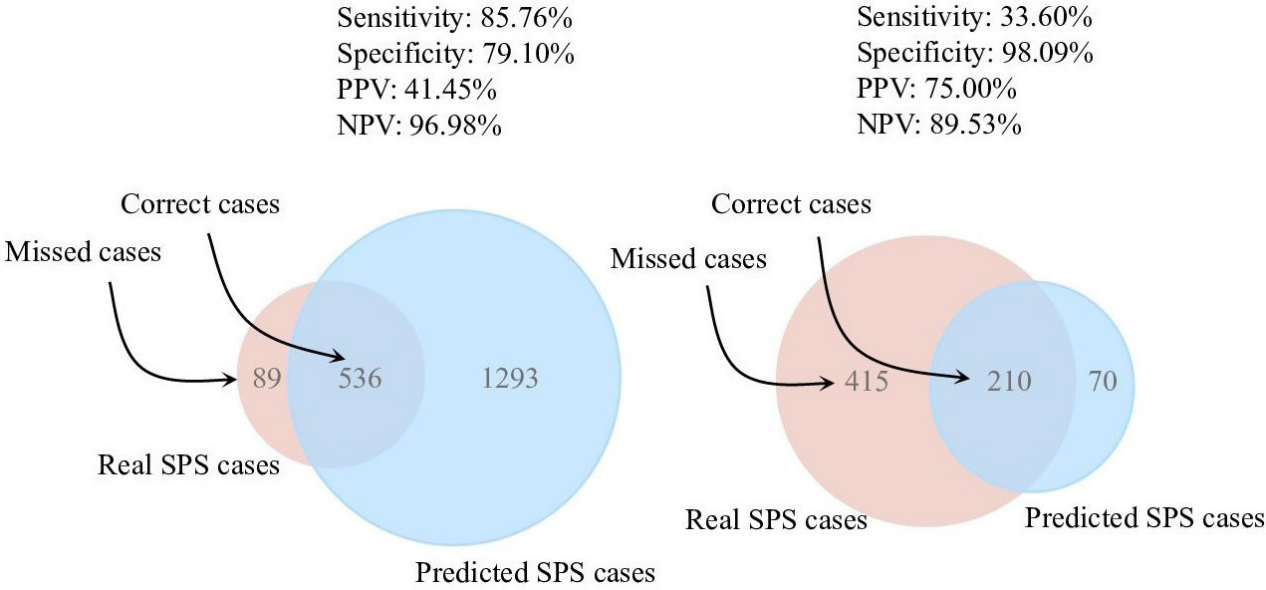
Results of XGBoost on test set. NPV, negative predictive value; PPV, positive predictive value.

XGBoost outperformed the other ML model (online supplemental table 3), achieving the highest AUC (0.89, 95% CI: 0.88 to 0.90), balanced accuracy (0.80) (figure 1) (Section 1 of the online supplemental appendix lists the nine ML methods, their settings and evaluation metrics. Section 2 provides the rationale for not adopting Lasso for feature selection in this study, as shown in online supplemental figure 3), and shows the missing data pattern in online supplemental figure 4). It also had strong discriminatory power (online supplemental figure 5) for t-SNE visualisation.

**Figure 2:**
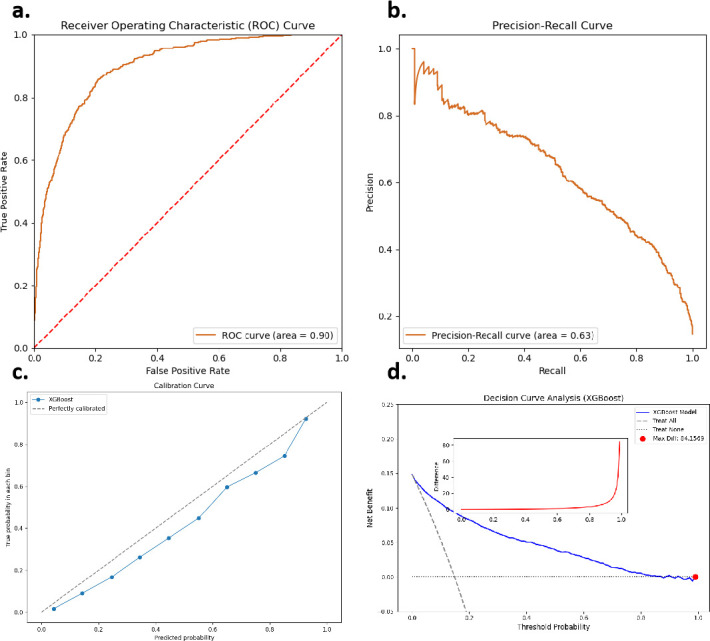
Depiction of the XGBoost performance predicting subclinical psychotic symptoms at two different thresholds. The probability threshold was 0.17 (left) and 0.76 (right). (A) Receiver Operating Characteristic curve, (B) precision–recall curve, (C) calibration curve and (D) decision curve.

The XGBoost model performed well at two thresholds (see figure 2). At a threshold of 0.17 (chosen for best balance of sensitivity and specificity), the model had 85.76% sensitivity, 79.10% specificity, 96.98% NPV and 41.45% PPV. At a threshold of 0.76, where NPV and PPV were balanced, the model showed a specificity of 98.09%, sensitivity of 33.60%, NPV of 89.53% and PPV of 75.00%. More threshold results are in online supplemental table 4.

The calibration curve was close to the ideal line, showing the model’s predictions were mostly accurate, with only a slight overestimation (see figure 2c). The model also gave a higher net benefit than always treating everyone at most thresholds, meaning it helped improve decisions and reduce unnecessary treatments. The red dot marks the threshold with the highest net benefit compared with treating all patients, which is useful for doctors (see figure 2d). Overall, these results show the model performed very well.

We tested the model on a separate external SPS dataset to see how well it works on new data. The XGBoost model got an AUC of 0.8865 and a balanced accuracy of 0.7752 (online supplemental figure 6), showing it is accurate and generalises well.

Online supplemental figure 7 shows the ROC curves for different k values of Synthetic Minority Over-sampling Technique, and the model performed well in all cases, showing stable and reliable results.

### Feature importance analysis

**Figure 3:**
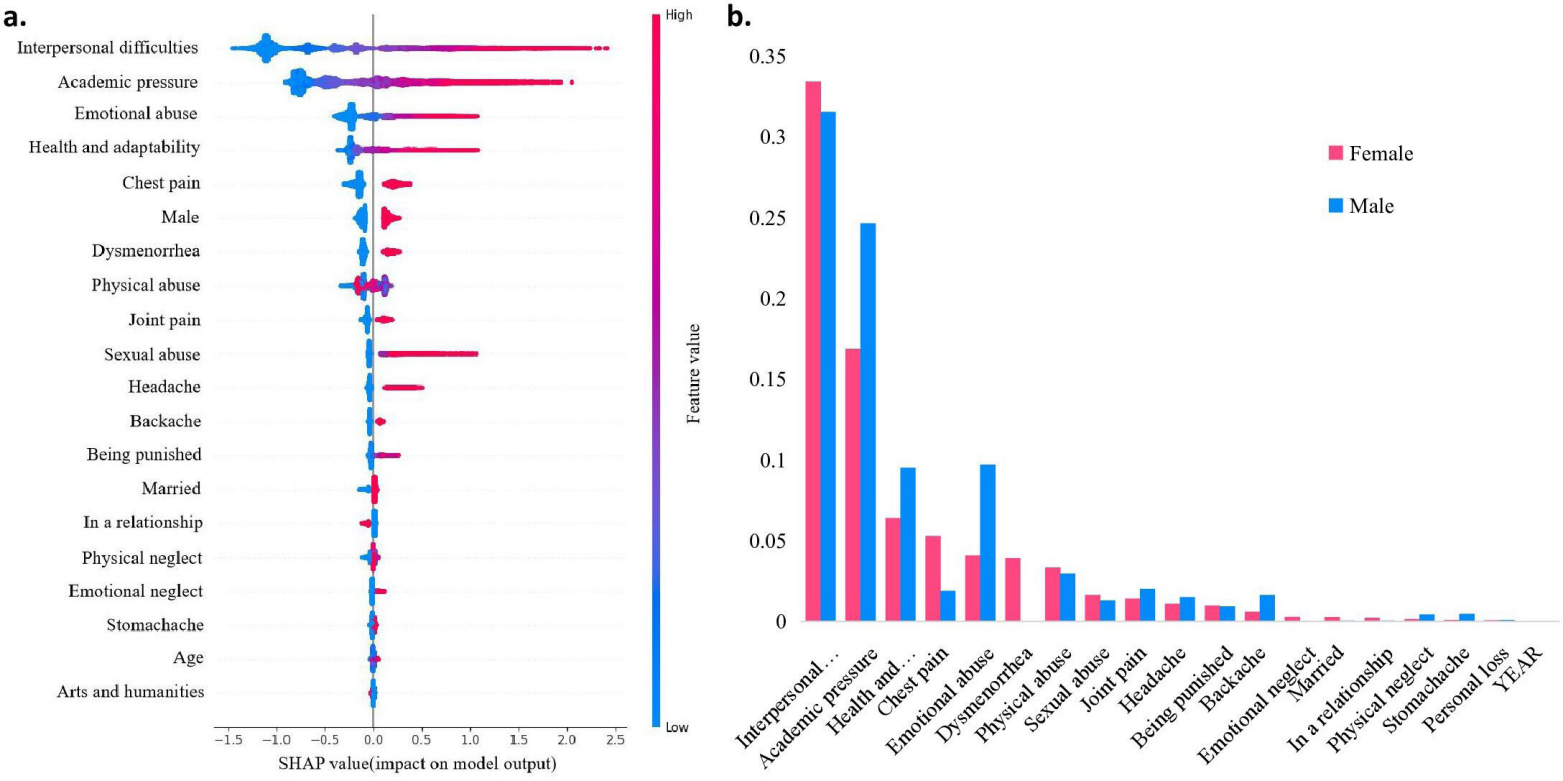
Summary SHAP plot. (A) Feature importance for SPS classification calculated by SHAP. (B) Influence of various features across gender. SHAP, Shapley Additive Explanation; SPS, subclinical psychotic symptom.

Figure 3a shows the 20 most important features for the model, ranked by SHAP values. Interpersonal difficulties were the strongest predictor of SPS, followed by academic pressure. Emotional abuse also contributed but was less important than the top two factors. Section 3 of the online supplemental appendix explains how we used SHAP to find important features and checked these results with other methods to make sure they are reliable. Online supplemental table 5 provides feature importance rankings from traditional methods. SHAP waterfall plots (online supplemental figure 8) show how each factor raises or lowers the predicted risk of SPS.

Both Gain and SHAP found that interpersonal difficulties and academic pressure are the main predictors of SPSs. However, SHAP also showed how less important features, like pain-related variables, can still strongly affect some individuals’ risk. This means that while some factors are important overall, other features may matter more for certain people.

### Gender differences in ML models

Gender analysis showed that different factors affect SPS in males and females (figure 3b). Academic pressure and emotional abuse had a bigger impact on males. In females, health issues like chest pain and dysmenorrhoea were more important. Interpersonal difficulties and academic pressure were top predictors for both genders, but their influence varied. These results suggest that gender should be considered when interpreting the model.

To better measure how gender influences SPS risk and reduce potential confounding, we used a Causal Forest model. The right-skewed distribution of Individual Treatment Effects suggests that gender impacts SPS risk for some individuals. The Average Treatment Effect was 0.04, indicating a slightly higher SPS risk in males after adjusting for other factors. This analysis offers a more detailed understanding of how gender moderates the effects of different risk factors (online supplemental figure 9).

### Mediation model

The analysis reveals a significant indirect effect (*β*=0.11, p<0.001), indicating that depressive symptoms mediate the relationship between sexual abuse and SPS. This finding supports the hypothesis that sexual abuse affects SPS both directly and indirectly through its impact on depressive symptoms (see online supplemental table 6 and figure 10).

## Discussion

Our ML models revealed strong associations between stressful life events, childhood trauma and SPS in undergraduates. This study, the first of its kind to use ML on a large sample to assess multiple SPS risk factors, confirmed established links such as abuse, social adversity and physical health issues. Notably, we identified two primary risk factors: interpersonal difficulties and academic stress, which significantly impacted SPS. Emotional abuse, though less influential, still contributed to SPS, highlighting the importance of addressing this often-overlooked form of maltreatment. Specifically, gender differences were also observed in the model. Academic pressure and emotional abuse had a stronger effect on males, while physical health issues like chest pain and dysmenorrhoea were more significant for females. These findings underscore the distinct ways in which various factors affect males and females.

This study underscores the critical role of interpersonal difficulties in the development of SPS, building on prior research that found young people with SPS often report more interpersonal challenges during adolescence, emphasising the significant impact of personal difficulties on SPS.^20^ Longitudinal twin cohort studies also highlight the protective role of interpersonal relationships in reducing the impact of poly-victimisation on SPS.^21^ Our findings align with a semistructured interview study, which showed that individuals at risk for psychosis often face significant interpersonal challenges.^22^ However, our study extends these findings by demonstrating that interpersonal difficulties are the most significant factors among various adverse life stressors and childhood traumas contributing to SPS. Studies suggest that psychosocial interventions focusing on interpersonal skills can aid rehabilitation in individuals with psychotic disorders,^23^ supporting our conclusion that addressing interpersonal difficulties is crucial in preventing SPS. Therefore, targeted psychological and social interventions addressing interpersonal challenges may help protect young people from SPS and its long-term consequences.

In line with previous research,^24^ we also observed that academic stress was another significant contributor to SPS among undergraduates. In the current education system in Mainland China, exam-performance-driven progression reaches from primary school to postgraduate studies. Young people have been facing increasingly fierce competition and peer pressure since childhood, and most likely throughout their careers. Interestingly, a cross-cultural study showed that the academic burden was highest in students from China who were the most stressed compared with students from the USA, Japan and South Korea.^25^ Such academic pressure on students in China may originate from their high-standard requirements, parents’ expectations and social values of praising the ‘tiger parenting’. These internal and external pressures stemming from intense competition can increase college students’ stress levels, lead to burnout and heighten their vulnerability to mental health problems. Our findings highlight academic stress as an important, yet previously overlooked, target for practitioners aiming to prevent or intervene in SPS among college students.

In our data, we found that emotional abuse, more than other forms of childhood trauma, is independently associated with SPS in this population. Emotional abuse, often under-recognised, refers to harmful interactions in the carer–child relationship that do not involve physical contact. Our results align with prior studies that emotional abuse was the only form of childhood trauma associated with SPS, possibly because emotional abuse occurs more frequently than other types of abuse subtypes,^26^ increasing the risk of SPS. From a neurodevelopmental perspective, emotional abuse can disrupt an individual’s hypothalamic–pituitary–adrenal axis,^27^ increasing the risk of developing SPS.

While our data show a significant association between sexual abuse and SPS, this effect diminished when other risk factors, such as academic stress and interpersonal difficulties, were included in the ML models. Although academic stress, interpersonal difficulties and emotional abuse are the primary contributors, the role of sexual abuse in SPS should not be overlooked.^28^ This may be because our analysis showed that depressive symptoms connect sexual abuse and SPS.

Our findings reveal gender-specific differences in the impact of various factors on SPS among undergraduates, offering new insights into the complexity of risk factors for males and females. Both academic pressure and emotional abuse emerged as prominent predictors for both genders, but these factors had a stronger influence on males, particularly academic pressure. This suggests that boys may experience greater stress and emotional abuse due to the high expectations placed on them in Chinese culture. Traditionally, men are expected to shoulder more responsibility, and society tends to look down on men who are unsuccessful in their careers as well as women who display excessive career ambition. For example, a study shows that in Chinese academia, men are often expected to achieve more and support their families, while less is expected of women.^29^ Therefore, cultural beliefs and traditional gender roles put extra pressure on boys, making these problems worse. These factors show that culture affects stress differently for male and female. For females, health issues like chest pain and period pain increase their risk of SPS. This means it’s important to pay attention to gender-specific health problems in future studies.

These gender differences show that we need different support strategies for male and female students. Future studies should pay attention to how gender affects SPS and why some issues, like academic pressure, may affect males more. Understanding the social, psychological and biological factors behind these differences in the future will help us develop more effective support programmes for all undergraduate students.

Despite using large samples and robust ML methods to explore risk factors for SPS among undergraduate students in Southwest China, several limitations must be considered when interpreting the results. First, assessments of childhood trauma and stress events rely on retrospective self-reports, which may be influenced by recall bias or social desirability effects. Future studies should incorporate both objective and subjective measures to strengthen the findings and provide further validation. Second, we did not assess substance use or mood/anxiety symptoms, which could be important covariates or mediators; these were excluded based on pilot study findings and expert input. Third, due to sensitivity concerns, mental illness diagnoses were not collected. Fourth, while we excluded participants who failed comprehension checks, some may have misinterpreted questions. Fifth, accuracy differences across the nine ML models were minimal, but the use of only XGBoost results may reflect model bias. Sixth, pain symptoms had low predictive value and were not retained in the final prediction model; this may limit the clinical applicability of our model in populations where such symptoms are common, such as individuals with chronic health conditions. Finally, as our study focused on university students from Southwest China within a limited age range, and given the relatively high non-response rate, there may be some bias in our results. Therefore, our findings should be interpreted with caution when considering their generalisability to university students nationwide or all Chinese youth. Furthermore, our model still requires further research and external validation to ensure its reliability and wider application.

Previous studies in Western countries have found that most people with SPS experienced childhood trauma or negative life events,^30^ but they did not build predictive models for SPS based on these factors, so our study offers new insights into what could affect SPS in this population. This can help future research and support programmes for students.

In conclusion, our study identified key risk factors for SPS in university students, using ML and large sample sizes to assess both childhood trauma and recent stressors. We confirmed known risk factors like domestic abuse and social adversity, but highlighted interpersonal difficulties and academic stress and emotional abuse as the most significant contributors. Gender differences were observed, with academic pressure and emotional abuse having a stronger impact on males, while health issues like chest pain and dysmenorrhoea were more significant for females, offering a gender-differentiated perspective on SPS risk and suggesting the need for gender-tailored interventions. Future studies should explore the mechanisms behind gender differences, track SPS risk factors longitudinally and include additional variables like substance use and mood/anxiety symptoms. Expanding this research across different cultural contexts will further refine these findings and improve intervention strategies.

It is also important to consider how cultural and psychosocial stressors may interact with neurodevelopmental pathways to increase the risk for SPS. Ongoing interpersonal difficulties, academic stress and emotional abuse within a high-pressure environment may disrupt the HPA axis,^27^ which links early stress to later mental health symptoms. This helps provide a biological explanation for our findings and suggests that both cultural and biological factors should be considered in future research. However, the specific ways cultural stress affects neurobiology and gender differences still need more study.

## Supplementary material

10.1136/bmjment-2025-301761 online supplemental file 1

## Data Availability

Data are available upon reasonable request.
