# Supplementary material for: Childhood trauma and recent stressors in predicting subclinical psychotic symptoms among Chinese university students in southwest China: a machine learning analysis within a gender-specific framework
Source: BMJ Ment Health. 2025 Jul 31;28(1):e301761. doi: 10.1136/bmjment-2025-301761 (PMC12314930; doi:10.1136/bmjment-2025-301761)
Supplement: online supplemental file 1 [file bmjment-28-1-s001.docx]

**Supplementary Materials for**

Childhood Trauma and Recent Stressors in Predicting Subclinical Psychotic Symptoms among Chinese University Students in Southwest China: A Machine Learning Analysis within a Gender-Specific Framework

**Sections 1 to 3:**

Section 1: A more detailed description of the machine learning methods used, the corresponding model hyperparameters, and the introduction of the evaluation metrics.
 Section 2: The experimental implementation process, ensuring the reproducibility of the experiment.
 Section 3: The theoretical basis and details of the SHAP analysis, supplemented by traditional machine learning feature importance analysis.

**Supplementary Table 1 to 6:**

Supplementary Table 1: Baseline Characteristics of Participants and Non-participants in the Survey

Supplementary Table 2. Sociodemographic and clinical psychological characteristics (N = 21,208)

Supplementary Table 3. Performance of nine machine learning models in 5-folds cross validation

Supplementary Table 4: Analysis of XGBoost model results under different thresholds.
 Supplementary Table 5: Importance rankings of each feature under different models' traditional feature importance algorithms.

Supplementary Table 6: A mediation model between sexual abuse and SPS with age and gender controlled.

**Supplementary Figure 1 to 10:**

Supplementary Figure 1. Inter-variable correlation Heatmap.

Supplementary Figure 2: VIF values of majority variables.

Supplementary Figure 3: Features selected through LASSO regularization.

Supplementary Figure 4: Missing pattern heatmap of all samples.

Supplementary Figure 5: t-SNE Visualization results on test set.

Supplementary Figure 6: ROC of XGBoost on external test set.

Supplementary Figure 7. ROC curves of XGBoost on the test set under different k values of SMOTE.

Supplementary Figure 8: Examples of Influence of features to specific samples.

Supplementary Figure 9. Distribution of Individual Treatment Effects (ITEs) and Average Treatment Effect (ATE) from the Causal Forest model assessing gender's moderating effect on SPS risk.

Supplementary Figure 10. Depressive symptoms mediated the relationship between childhood sexual abuse and SPS

**Appendix References**

**Section 1. Method**

Machine Learning Models

In this work, we employed six machine learning methods: the Extreme Gradient Boosting (XGBoost), Random Forest (RF), Logistic Regression (LR), Gradient Boosting Tree (GBT), Support Vector Machine (SVM), and Multilayer Perceptron (MLP). Below is a brief description of each model and its hyper-parameters:

XGBoost: XGBoost is a scalable, parallelized gradient boosting tool that enhances the predictive accuracy of decision trees [1]. It iteratively generates models, and the sum of these models constitutes the final output. XGBoost optimizes the loss function using a second-order Taylor expansion, incorporating second-order derivative information to greedily determine whether to split nodes. The hyper-parameters for XGBoost were configured as follows: learning_rate = 0.02, n_estimators = 1000 (the number of trees in the model), max_depth = 20, gamma = 9, subsample = 0.6, random_state = 50, reg_lambda = 0.7, and booster = 'gbtree', with other parameters set to their default values. We optimized the XGBoost model by tuning key hyperparameters to maximize ROC. Specifically, for each tree model, the learning rate was searched over [0.3,0.1,0.03,0.02,0.01,0.001,0.0001][0.3, 0.1, 0.03, 0.02, 0.01, 0.001, 0.0001][0.3,0.1,0.03,0.02,0.01,0.001,0.0001]; the number of estimators included values of 10, 50, 100, 150, 200, 250, 300, 350, 400, 450, 500, 1000, 1100, and 1200; and the maximum tree depth was explored across 0, 4, 5, 6, 7, 8, 9, 10, 16, 24, and 32.

Random Forest (RF): RF is an ensemble learning algorithm that aggregates predictions from multiple decision trees to produce more reliable results [2]. Each tree is generated by randomly sampling subsets from the original training data, with nodes and branches constructed using randomly selected feature subsets. In this analysis, we used Random Forest with hyper-parameters provided by the scikit-learn library. The key default parameters were as follows: n_estimators: 1000, criterion: 'gini' (used for measuring the quality of splits), min_samples_split: 5, max_features: ‘sqrt’ and bootstrap set ‘True’.

Gradient Boosting Tree (GBT): GBT is an ensemble learning technique that builds a series of decision trees sequentially, where each tree is trained to correct the errors made by the previous ones (Friedman, 2001). Unlike Random Forest, where trees are trained independently, GBT focuses on boosting weak learners iteratively by minimizing the loss function. At each step, a new tree is added to improve the model by fitting the residual errors of the prior trees. The final model is the weighted sum of all the trees, which makes GBT effective in capturing complex patterns in the data and achieving high predictive accuracy. We employed GBT with hyper-parameters as: n_estimators: 500, learning_rate: 0.01, max_depth: 12, subsample: 0.6 and loss function as 'deviance' (for classification, this corresponds to using a logistic regression loss function)

Logistic Regression (LR): Logistic Regression is a generalized linear model used for binary classification. It estimates model parameters (such as weights and biases in the sigmoid function) based on the training data to predict the probability of a given class with all the hyper-parameters set as default using L2 penalty.

Support Vector Machine (SVM): SVM aims to maximize the margin between different classes by finding the optimal hyperplane in the feature space, formulated as a convex quadratic programming problem [3]. For our task, we used a kernelized SVM with a Radial Basis Function (RBF) kernel to capture the non-linear relationships in the data.

Multilayer Perceptron (MLP): MLP is a type of feedforward artificial neural network that maps input vectors to output vectors. It updates the network's weights by calculating the loss between the predicted and target values and then propagating this loss backward through the network. In our implementation, we utilized an MLP with two hidden layers, and each containing ten units while 'ReLU' was set as the Activation Function and Binary Cross-Entropy as loss function.

Naive Bayes (NB): Naive Bayes is a family of probabilistic classifiers based on Bayes’ theorem with the “naive” assumption of conditional independence between features. It computes the posterior probability of each class given the input features and assigns the class with the highest probability. We used the Gaussian variant of Naive Bayes, which assumes normally distributed features.

CatBoost: CatBoost is a gradient boosting algorithm developed to handle categorical variables efficiently without explicit preprocessing. It uses ordered boosting and target statistics to reduce overfitting and improve generalization. In our implementation, CatBoost was used with default parameters, leveraging its native handling of categorical features and robustness to overfitting.

AdaBoost: AdaBoost (Adaptive Boosting) is an ensemble learning method that combines multiple weak learners—typically decision stumps—into a strong classifier. It iteratively adjusts the weights of training samples based on their classification errors, placing more emphasis on hard-to-classify instances. We used AdaBoost with decision trees as base estimators and default learning settings.

KNN: K-Nearest Neighbors (KNN) is a non-parametric, instance-based learning algorithm that classifies data points based on the majority class among their K closest neighbors in the feature space. In this study, we used KNN with default settings and employed Euclidean distance as the similarity measure. KNN makes no strong assumptions about the data distribution and is effective in capturing local patterns. However, it can be sensitive to outliers and may suffer from the "curse of dimensionality" in high-dimensional spaces.

Evaluation Metrics

The performance of these models were evaluated using several key metrics, including Sensitivity, Specificity, Balanced Accuracy, Positive Predictive Value (PPV), Negative Predictive Value (NPV), and Area Under the Curve (AUC). First, we introduce the confusion matrix, where the terms are defined as TP (True Positive), TN (True Negative), FP (False Positive), and FN (False Negative). Thus, these metrics are defined as follows:

Sensitivity (Recall or True Positive Rate, TPR): Measures the proportion of actual positive cases that the model correctly identifies. It is calculated as:

$$Sensitivity=\frac{\mathrm{TP}}{TP+FN}$$

Specificity (True Negative Rate, TNR): Reflects the proportion of actual negative cases that are correctly identified by the model. The formula is:

$$Specificity=\frac{\mathrm{TN}}{TN+FP}$$

Balanced Accuracy: This metric provides an average of Sensitivity and Specificity, offering a more balanced assessment when dealing with imbalanced datasets. It is calculated as:

$$Balanced Accuracy=\frac{Sensitivity+Specificity}{2}$$

Positive Predictive Value (PPV or Precision): Indicates the proportion of positive predictions that are accurate. It is computed as

$$PPV=\frac{\mathrm{TP}}{TP+FP}$$

Which highlighting the model’s reliability when predicting positive outcomes.

Negative Predictive Value (NPV): Reflects the proportion of negative predictions that are accurate, calculated as:

$$NPV=\frac{\mathrm{TN}}{TN+FN}$$

A higher NPV indicates that negative predictions are more likely to be correct.

Area Under the Curve (AUC): Represents the area under the Receiver Operating Characteristic (ROC) curve, which plots Sensitivity against 1 - Specificity across different thresholds. The AUC value ranges from 0 to 1, where a higher AUC indicates better model performance and its ability to discriminate between positive and negative classes.

**Section 2. Training Details**

The variance threshold method is an unsupervised feature selection technique that selects variables based on their variance, without considering their relationship with the target variable. The core idea is that features with low variance exhibit minimal variation within the data and thus contain limited information, making them suitable for exclusion. The process involves: first, cleaning the data, then calculating the variance for each feature. A variance threshold is set (In this work, the threshold was set as 0.01) based on domain knowledge or cross-validation results, retaining features with variance above this threshold.

During the data pre-processing stage, we set the random state for data splitting to 2023 to ensure that all models were trained and validated on the same data. The pre-processing steps were consistent across all five models. Specifically, we applied one-hot encoding to categorical variables, as classifiers typically assume data is continuous and ordered. This step was necessary to map discrete feature values into Euclidean space, ensuring that each discrete value corresponds to a unique point.

In our dataset, participants were required to complete all survey items before submission, resulting in no observed missing values. We confirmed this through a missing pattern heatmap. Nonetheless, to ensure robustness, we implemented mean imputation in our preprocessing code as a precautionary measure. This had minimal impact on the results. A brief analysis of the missing data pattern and a sensitivity note have been added to improve methodological transparency.

To complement our analysis, we conducted LASSO-based feature selection. However, given the relatively small number of predictors retained after excluding variables directly used in SPS calculation, strict application of LASSO was not necessary. Moreover, models such as XGBoost and LightGBM incorporate regularization-based feature selection, which helps mitigate redundancy. Considering our goal of exploring associations between psychosocial and behavioral factors and SPS, we retained all theory-driven variables in the main analysis to avoid omitting potentially relevant factors. The LASSO results are reported in the Supplementary Materials as an additional reference.

Due to the potential bias that some models may exhibit when handling imbalanced datasets, which can affect the fairness of model comparisons, we implemented the SMOTE (Synthetic Minority Over-sampling Technique) algorithm [5] with 5 neighbors on the training set to address class imbalance. To further evaluate the impact of different k values on model performance, we conducted additional comparison experiments that illustrate the changes in test set ROC curves across different k settings. Additionally, we have supplemented the class ratios before and after SMOTE and provided a visual comparison of the distributions，suggesting that k=5 is the most effective setting for improving model performance. Additionally, we handled missing values using mean imputation for continuous variables and mode imputation for categorical variables. Outliers were identified using the interquartile range (IQR) method and adjusted accordingly. After splitting the dataset, we standardized the features but skipped this step for ensemble tree models, which do not require standardization. Each model was fine-tuned using a grid search to identify the optimal hyper-parameters, with all search ranges set within reasonable bounds.

**Section 3. Feature Analysis Methods**

We employed multiple methods to calculate feature importance, with SHAP (SHapley Additive exPlanations) being one of them. SHAP offers a model-agnostic explanation by assigning an importance value to each feature in a prediction sample. Specifically, for each prediction, the model produces a predicted value, and SHAP assigns a numerical contribution to each feature in that sample [6].

SHAP Value

SHAP (SHapley Additive exPlanations) values were calculated to explain the contribution of each feature to the model’s predictions. SHAP values are derived based on cooperative game theory, which calculates the marginal contribution of each feature by averaging the impact over all possible feature combinations. This ensures a fair distribution of feature importance, providing both local and global interpretability. Specifically, SHAP values for each feature quantify how much that feature shifts the model’s output from the average prediction, offering a consistent way to interpret complex models like XGBoost. SHAP values are calculated based on the concept of Shapley values from cooperative game theory, aiming to measure the contribution of each feature to the model's prediction. For a given feature i, the Shapley value $\phi_{i}$ could be calculated by：

$$\phi_{i}=\sum_{S\subseteq N\backslash\{i\}} \frac{|S|!(|N|-|S|-1)!}{|N|!}[f(S\cup\{i\})-f(S)]$$

In this formula, N was defined as feature set, S denoted subset of features, excluding features i, and f(S) is the set of features in S on the model predictions. This formula calculates the average marginal contribution of feature iii across all possible subsets.

SHAP waterfall plot

To visualize the impact of SHAP values on individual predictions, we utilized SHAP waterfall plots. The SHAP waterfall plot starts from the base value (the mean model output for the training dataset) and progressively adds or subtracts each feature’s SHAP value according to its contribution to the final prediction. Specifically, the plot starts from the base value and then progressively shows how each feature's SHAP value contributes to the final prediction. The base value $E(f(X)$represents the mean output of the model over the training data:

E$(f(X))=\frac{1}{n}\sum_{j=1}^{n} f(x_{j})$

Where $E(f(X))$the initial prediction is value, and $x_{j}$ represents each training sample. The cumulative impact of each feature$i$'s SHAP value $\phi_{i}$​ can be expressed as:

$$\hat{y}=E(f(X))+\sum_{i=1}^{M} \phi_{i}$$

Where $\hat{y}$the final is predicted value, $M$ is the total number of features for the sample, and $\sum_{i=1}^{M} \phi_{i}$represents the sum of all feature contributions to the prediction. The SHAP waterfall plot displays each feature’s SHAP value as horizontal bars, allowing us to see which features increase or decrease the predicted value, helping to interpret the model’s decision-making for a particular sample.

Traditional Feature importance

In addition, we calculated traditional feature importance for these models. In ensemble tree models, feature importance is measured through 'gain,' a generalized concept of information gain. In this context, 'gain' refers to the average improvement in the objective function when a node is split based on a particular feature. Specifically, for a node j in a tree model, the split gain is expressed as:

$$Gain(j)=\frac{Impurity(parent)-Impurity(left)\times Samples(left)-Impurity(right)\times Samples(right)}{Samples(parent)}$$

Where Impurity (parent) is the impurity of the parent node (typically Gini impurity or mean squared error). Impurity (left) and Impurity (right) are the impurities of the left and right child nodes, respectively. Samples (left) and Samples (right) are the sample counts in the left and right child nodes, respectively. Samples (parent) is the sample count in the parent node. The gain of a node represents the reduction in impurity achieved by splitting the node.

For Logistic Regression, a linear model, feature importance was derived from the absolute values of the coefficients (|w|) associated with each feature. The larger the coefficient, the greater its impact on the final prediction, with weights estimated using maximum likelihood estimation.

It is well known that different models calculate feature importance in various ways, which can lead to different results on the same dataset. However, in our case, despite the differences in calculation methods, there were significant commonalities in the feature importance rankings across the models. As shown in Supplementary Table 2, interpersonal difficulties, academic pressure, and experiences of psychological maltreatment in childhood consistently demonstrated a strong correlation with SPS, thereby strongly supporting our conclusions.

**Supplementary Tables**

Supplementary Table.1. Baseline Characteristics of Participants and Non-participants in the Survey

| Characteristic | Participants  (n=21534) | Non-participants  (n=10068) | P value |
| --- | --- | --- | --- |
| Age | 19.71 ± 2.33 | 19.70 ± 2.71 | 0.771 |
| Gender |  |  |  |
| Male | 9497 (44.78%) | 4560 (43.87%) | 0.130 |
| Famale | 11711 (55.22%) | 5834 (56.13%) |  |
| Major |  |  |  |
| Engineering | 6433 (30.33%) | 3223 (31.01%) | 0.226 |
| Science | 5948 (28.05%) | 3024 (29.09%) | 0.054 |
| Medicine | 2675 (12.61%) | 1211 (11.65%) | 0.015 |
| Arts and humanities | 6152 (29.01%) | 2937 (28.26%) | 0.170 |
| University year | 2.92 ± 1.03 | 2.92 ± 1.02 | 0.894 |

Abbreviations: Continuous variables were analyzed using t-test (Mean ± SD), while categorical variables were analyzed using $\chi^{2}$ test.

Supplementary Table 2. Sociodemographic and clinical psychological characteristics (N = 21,208)

| Characteristic | Overall (n=21208) | SPS | | $\chi^{2}$ | FDR p |
| --- | --- | --- | --- | --- | --- |
|  |  | Positive (n=3187) | Negative (n=18021) |  |  |
| Age |  |  |  |  |  |
| <18 | 991 (4.7%) | 181 (6.6%) | 810 (4.5%) | 82.80 | <0.001 |
| 18-20 | 15864 (74.8%) | 2423 (76.0%) | 13441 (74.6%) |  |  |
| >20 | 4353 (20.5%) | 583 (18.3%) | 3770 (20.9%) |  |  |
| Sex |  |  |  |  |  |
| Female | 11711 (55.2%) | 1730 (54.3%) | 9981 (55.4%) | 1.29 | 0.277 |
| Male | 9497 (44.8) | 1457 (45.7%) | 8040 (44.6%) |  |  |
| LBC |  |  |  |  |  |
| Yes | 5206 (24.5%) | 1041 (32.7%) | 4165 (23.1%) | 132.89 | <0.001 |
| No | 16002 (75.5%) | 2146 (67.3%) | 13856 (76.9%) |  |  |
| Civil status |  |  |  |  |  |
| In a relationship | 5182 (24.4%) | 636 (20.0%) | 4546 (25.2%) | 40.45 | <0.001 |
| Single | 16026 (75.6%) | 2551 (80.0%) | 13475(74.8%) |  |  |
| University year |  |  |  |  |  |
| 1 | 2377 (11.2%) | 289 (9%) | 2088 (11.6%) | 135.33 | <0.001 |
| 2 | 5060(23.9%) | 668 (21.0%) | 4392 (24.4%) |  |  |
| 3 | 5612 (26.5%) | 711 (22.3%) | 4901 (27.2%) |  |  |
| 4 | 8159 (38.4%) | 1519 (47.7%) | 6640 (36.8%) |  |  |
| Major |  |  |  |  |  |
| Engineering | 6433 (30.3%) | 1009 (31.7%) | 5424 (30.1%) | 3.05 | 0.095 |
| Science | 5948 (28.0%) | 887 (27.8%) | 5061 (28.1%) |  |  |
| Arts and humanities | 6152 (29.0%) | 887 (27.8%) | 5265 (29.2%) |  |  |
| Medicine | 2575 (12.1%) | 404 (12.7%) | 2271 (12.6%) |  |  |
| Hermental |  |  |  |  |  |
| Yes | 457 (2.2%) | 139 (4.4%) | 318 (1.8%) | 85.39 | <0.001 |
| No | 20751 (97.8) | 3048 (95.6%) | 17703 (98.2%) |  |  |
| Childhood Abuse |  |  |  |  |  |
| Emotional abuse (≥13) | 1154 (5.4%) | 594 (18.6%) | 560 (3.1%) | 2427.75 | <0.001 |
| Emotional neglect (≥15) | 239 (1.1%) | 123 (3.8%) | 116 (0.6%) | 1054.86 | <0.001 |
| Sexual abuse (≥8) | 1111 (5.2%) | 446 (13.9%) | 665 (3.6%) | 903.71 | <0.001 |
| Physical abuse (≥10) | 13099 (62.8%) | 2078 (65.2%) | 11021 (61.2%) | 1100.44 | <0.001 |
| Physical neglect (≥10) | 9275 (43.7%) | 1308 (41.0%) | 7966 (44.2%) | 695.06 | <0.001 |
| ASLEC Score (Mean±SD） |  |  |  |  |  |
| Interpersonal difficulties |  |  |  |  |  |
| Low | 6696 (31.6%) | 125 (3.9%) | 6571 (36.5%) | 5728.03 | <0.001 |
| Medium | 10985 (51.8%) | 1286 (40.4%) | 9699 (53.8%) |  |  |
| High | 3527 (16.6%) | 1776 (55.7%) | 1751 (9.7%) |  |  |
| Academic pressure |  |  |  |  |  |
| Low | 3634 (17.1%) | 43 (1.3%) | 3591 (19.9%) | 5113.86 | <0.001 |
| Medium | 14543 (68.6%) | 1600 (50.2%) | 12943 (71.8%) |  |  |
| High | 3031 (14.3%) | 1544 (48.4%) | 1487 (8.3%) |  |  |
| Being punished |  |  |  |  |  |
| Low | 14329 (67.6%) | 1178 (37.0%) | 13151 (73.0%) | 3022.49 | <0.001 |
| Medium | 4964 (23.4%) | 1045 (32.8%) | 3919 (21.7%) |  |  |
| High | 1915 (9.0%) | 964 (30.2%) | 951 (5.3%) |  |  |
| Personal loss |  |  |  |  |  |
| Low | 14483 (68.3%) | 1448 (45.4%) | 13035 (74.0%) | 1523.71 | <0.001 |
| Medium | 3899 (18.4%) | 724 (22.7%) | 3175 (17.6%) |  |  |
| High | 2826 (13.3%) | 1015 (31.8%) | 1811 (10.0%) |  |  |
| Health and adaptability |  |  |  |  |  |
| Low | 6211 (29.3%) | 217 (6.8%) | 5994 (33.3%) | 3808.67 | <0.001 |
| Medium | 12678 (59.8%) | 1771 (55.6%) | 10907 (60.5%) |  |  |
| High | 2319 (10.9%) | 1199 (37.6%) | 1120 (6.2%) |  |  |
| Pain |  |  |  |  |  |
| Stomach | 9092 (42.9%) | 1971 (61.8%) | 7058 (39.2%) | 568.82 | <0.001 |
| Backache | 5253 (24.8%) | 1360 (42.7%) | 3893 (21.6%) | 644.10 | <0.001 |
| Joint pain | 5910 (27.9%) | 1589 (49.9%) | 4321(24.0%) | 901.13 | <0.001 |
| Dysmenorrhea | 6179 (29.1%) | 1635(51.3%) | 4544 (25.2%) | 891.35 | <0.001 |
| Headache | 1748 (8.2%) | 669 (21.0%) | 1079 (6.0%) | 804.13 | <0.001 |
| Chest pain | 6966 (32.8%) | 1932 (60.6%) | 5034 (27.9%) | 1310.3 | <0.001 |

Abbreviations: LBC, left behind children; SPS, subclinical psychotic symptoms; $\chi^{2}$ indicates the strength of association with SPS status; higher values suggest stronger associations. FDR p-values control for false positives across multiple tests.

Supplementary Table. 3. Performance of nine machine learning models in 5-folds cross validation

| Model | Train AUC | Test AUC | Balanced Acc | AUPRC | Brier | p Value |
| --- | --- | --- | --- | --- | --- | --- |
| XGBoost | 0.9163 | 0.8922 (0.8790-0.9043) | 0.8043 | 0.6377 | 0.0842 | / |
| GBT | 0.8987 | 0.8872 (0.8775-0.9025) | 0.7449 | 0.6267 | 0.0921 | 0.040 |
| LR | 0.8940 | 0.8723 (0.8691-0.8947) | 0.7985 | 0.6181 | 0.0853 | 0.030 |
| MLP | 0.9102 | 0.8665 (0.8508-0.8812) | 0.7875 | 0.5953 | 0.1446 | <0.001 |
| RF | 0.8924 | 0.8847 (0.8709-0.8978) | 0.7263 | 0.6082 | 0.0907 | 0.007 |
| SVM | 0.9401 | 0.8625 (0.8478-0.8766) | 0.7692 | 0.5274 | 0.0969 | <0.001 |
| Naive Bayes | 0.8875 | 0.8112 (0.7925-0.8292) | 0.7644 | 0.5308 | 0.1470 | <0.001 |
| CatBoost | 0.9925 | 0.8865 (0.8747-0.8969) | 0.7353 | 0.6145 | 0.0857 | 0.033 |
| AdaBoost | 0.9514 | 0.8662 (0.8534-0.8773) | 0.7752 | 0.5270 | 0.2391 | <0.001 |
| KNN | 0.9062 | 0.8244 (0.8091-0.8398) | 0.7611 | 0.5319 | 0.1646 | <0.001 |

Abbreviations: RF, Random Forest; GBT, Gradient Boosting Trees; LR, Logistic Regression; SVM, Support Vector Machines; MLP, Multilayer Perceptron; KNN, k-nearest neighbors

Supplementary Table.4. Metrics under different thresholds.

| Threshold | Sensitivity | 95% CI | Specificity | 95% CI | 95% CI | 95% CI |
| --- | --- | --- | --- | --- | --- | --- |
| >0.014 | 99.84 | 99.1 - 100.0 | 16.56 | 15.4 - 17.8 | 1.2 - 1.2 | 0.001 - 0.07 |
| >0.165 | 85.60 | 82.6 - 88.3 | 79.10 | 77.7 - 80.4 | 3.8 - 4.4 | 0.2 - 0.2 |
| >0.251 | 74.88 | 71.3 - 78.2 | 86.51 | 85.4 - 87.6 | 5.1 - 6.1 | 0.3 - 0.3 |
| >0.305 | 69.28 | 65.5 - 72.9 | 89.13 | 88.1 - 90.1 | 5.7 - 7.1 | 0.3 - 0.4 |
| >0.354 | 63.84 | 59.9 - 67.6 | 91.10 | 90.1 - 92.0 | 6.4 - 8.1 | 0.4 - 0.4 |
| >0.400 | 60.00 | 56.0 - 63.9 | 92.56 | 91.7 - 93.4 | 7.1 - 9.2 | 0.4 - 0.5 |
| >0.450 | 55.04 | 51.0 - 59.0 | 93.81 | 93.0 - 94.6 | 7.7 - 10.3 | 0.4 - 0.5 |
| >0.500 | 51.68 | 47.7 - 55.7 | 95.27 | 94.5 - 95.9 | 9.3 - 12.9 | 0.5 - 0.6 |
| >0.543 | 48.00 | 44.0 - 52.0 | 96.10 | 95.4 - 96.7 | 10.3 - 14.8 | 0.5 - 0.6 |
| >0.630 | 40.96 | 37.1 - 44.9 | 97.43 | 96.9 - 97.9 | 12.8 - 19.9 | 0.6 - 0.6 |
| >0.661 | 37.60 | 33.8 - 41.5 | 97.73 | 97.2 - 98.2 | 13.1 - 21.0 | 0.6 - 0.7 |
| >0.702 | 33.60 | 29.9 - 37.5 | 98.09 | 97.6 - 98.5 | 13.6 - 22.8 | 0.6 - 0.7 |
| >0.731 | 30.56 | 27.0 - 34.3 | 98.29 | 97.8 - 98.7 | 13.6 - 23.4 | 0.7 - 0.7 |
| >0.778 | 25.12 | 21.8 - 28.7 | 98.95 | 98.6 - 99.3 | 17.0 - 33.7 | 0.7 - 0.8 |
| >0.801 | 23.04 | 19.8 - 26.5 | 99.03 | 98.7 - 99.3 | 16.6 - 34.1 | 0.7 - 0.8 |
| >0.826 | 20.00 | 16.9 - 23.4 | 99.14 | 98.8 - 99.4 | 15.9 - 34.3 | 0.8 - 0.8 |
| >0.867 | 15.04 | 12.3 - 18.1 | 99.45 | 99.1 - 99.7 | 16.9 - 43.7 | 0.8 - 0.9 |
| >0.901 | 10.56 | 8.3 - 13.2 | 99.78 | 99.6 - 99.9 | 23.0 - 99.0 | 0.9 - 0.9 |
| >0.944 | 4.00 | 2.6 - 5.8 | 99.94 | 99.8 - 100.0 | 17.2 - 304.7 | 0.9 - 1.0 |

Supplementary Table 5. Ranked feature importance for XGBoost, RF, GBT,and MLP.

|  | XGBoost | LG | RF | GBT | Ave. |
| --- | --- | --- | --- | --- | --- |
| Interpersonal difficulties | 1 | 1 | 1 | 1 | 1 |
| Academic pressure | 2 | 2 | 2 | 2 | 2 |
| Health and adaptability | 3 | 4 | 4 | 4 | 3.75 |
| Emotional abuse | 4 | 3 | 5 | 3 | 3.75 |
| Chest pain | 5 | 5 | 13 | 6 | 7.25 |
| Joint pain | 7 | 6 | 14 | 7 | 8.5 |
| Being punished | 6 | 20 | 7 | 5 | 9.5 |
| Dysmenorrhea | 9 | 7 | 15 | 8 | 9.75 |
| Sexual abuse | 16 | 10 | 11 | 11 | 12 |
| Personal loss | 21 | 11 | 9 | 13 | 13.5 |
| Physical abuse | 11 | 32 | 3 | 9 | 13.75 |
| Emotional neglect | 10 | 26 | 10 | 10 | 14 |
| Physical neglect | 19 | 16 | 6 | 16 | 14.25 |
| Headache | 8 | 13 | 19 | 23 | 15.75 |
| YEAR | 26 | 15 | 12 | 12 | 16.25 |
| Backache | 14 | 21 | 16 | 17 | 17 |
| Male | 20 | 8 | 26 | 18 | 18 |
| AGE | 22 | 31 | 8 | 14 | 18.75 |
| Married | 13 | 14 | 29 | 21 | 19.25 |
| In a relationship | 15 | 12 | 30 | 20 | 19.25 |
| Female | 26 | 8 | 25 | 22 | 20.25 |
| Stomachache | 23 | 27 | 17 | 15 | 20.5 |
| Arts and humanities | 12 | 25 | 22 | 27 | 21.5 |
| Science | 17 | 28 | 19 | 28 | 23 |
| Left behind children | 26 | 29 | 18 | 19 | 23 |
| Engineering | 26 | 24 | 21 | 26 | 24.25 |
| Single | 26 | 17 | 32 | 28 | 25.75 |
| HER | 25 | 19 | 31 | 28 | 25.75 |
| Medicine | 26 | 23 | 27 | 28 | 26 |

Importance ranging from 1 (most important) to 31 (less important).

ª Rank based on traditional feature importance.

^b^ No Conventional methods for MLP and SVM to calculate feature importance.

Supplementary Table 6. A mediation model between sexual abuse and SPS with age and gender controlled.

| Path | Effect | SE | *p* | 95% CI |
| --- | --- | --- | --- | --- |
| Sexual abuse (X)→Depressive symptoms(M) →SPS (Y) | | | | |
| Total effect of X on Y | 0.23 | 0.007 | <0.001 | 0.22-0.25 |
| Direct effect of X on Y | 0.12 | 0.005 | <0.001 | 0.11-0.14 |
| Indirect effect of X on Y | 0.11 | 0.009 | <0.001 | 0.09-0.13 |
| X→M | 0.17 | 0.007 | <0.001 | 0.16-0.18 |
| M→Y | 0.65 | 0.005 | <0.001 | 0.64-0.66 |

Abbreviations: SPS, subclinical psychotic symptoms; CI, confidence interval

**Supplementary Figures**


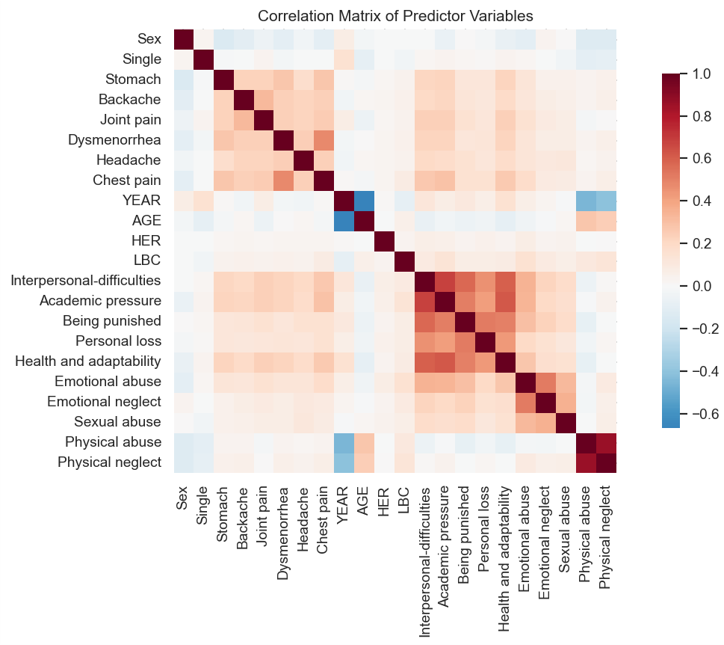


Supplementary Figure 1. Inter-variable correlation Heatmap.


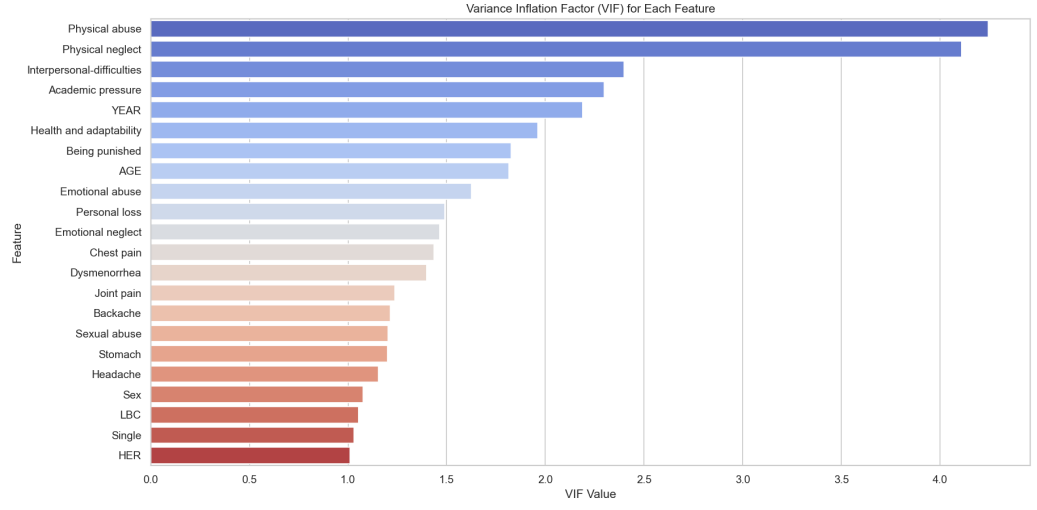


Supplementary Figure 2. VIF values of majority variables

.
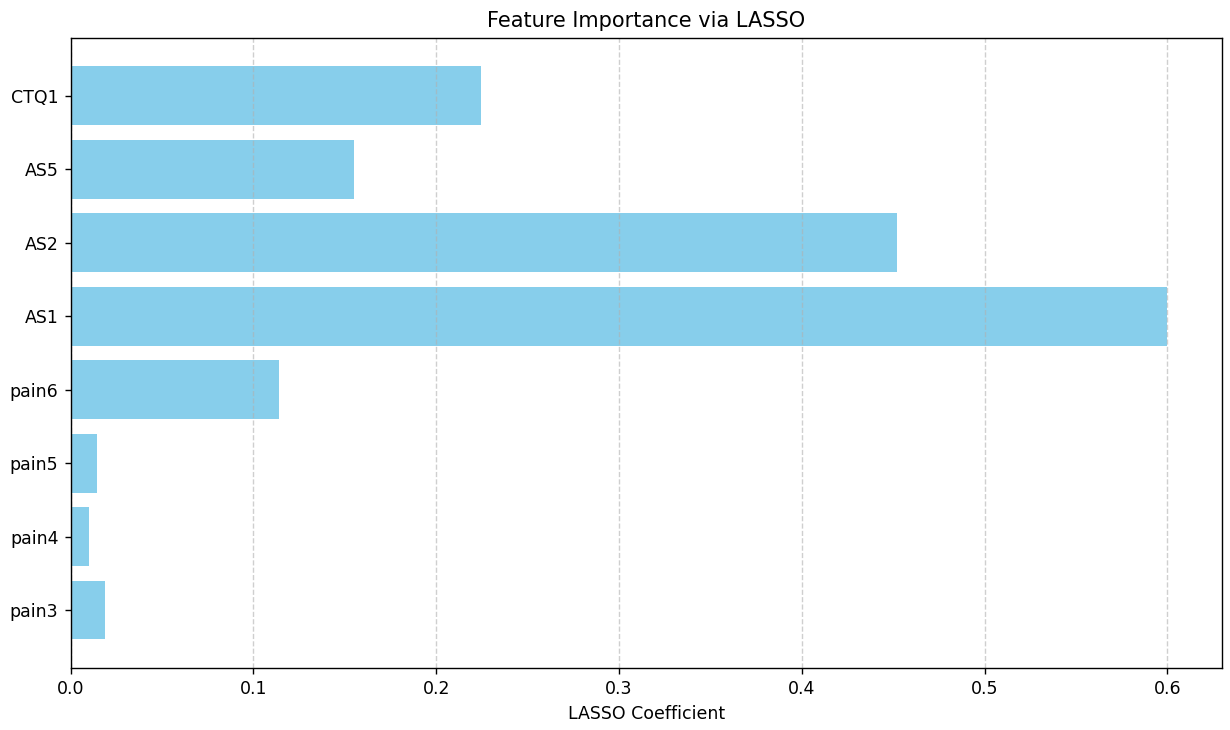


Supplementary Figure 3. Features retained after LASSO selection.


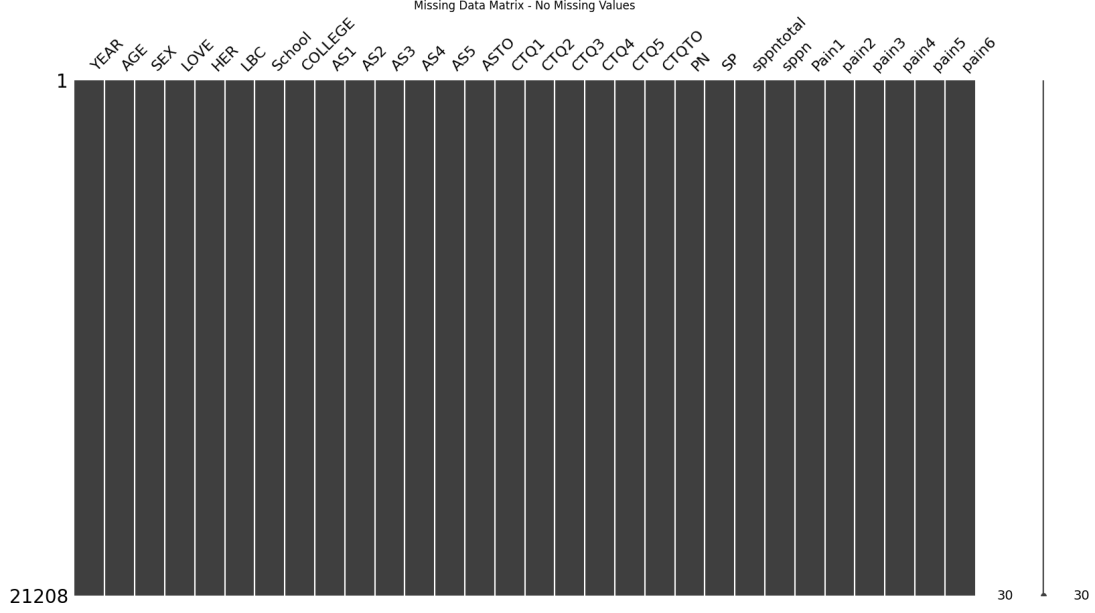


Supplementary Figure 4. Missing pattern heatmap of all samples.


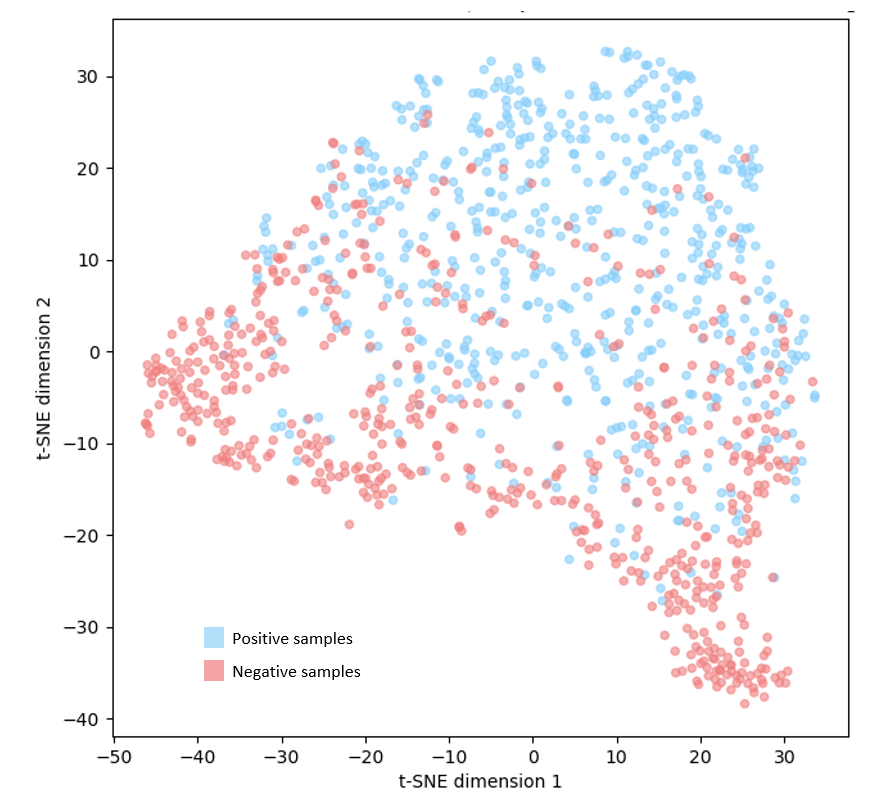


Supplementary Figure 5. t-SNE Visualization Embedding of features selected in XGBoost model.


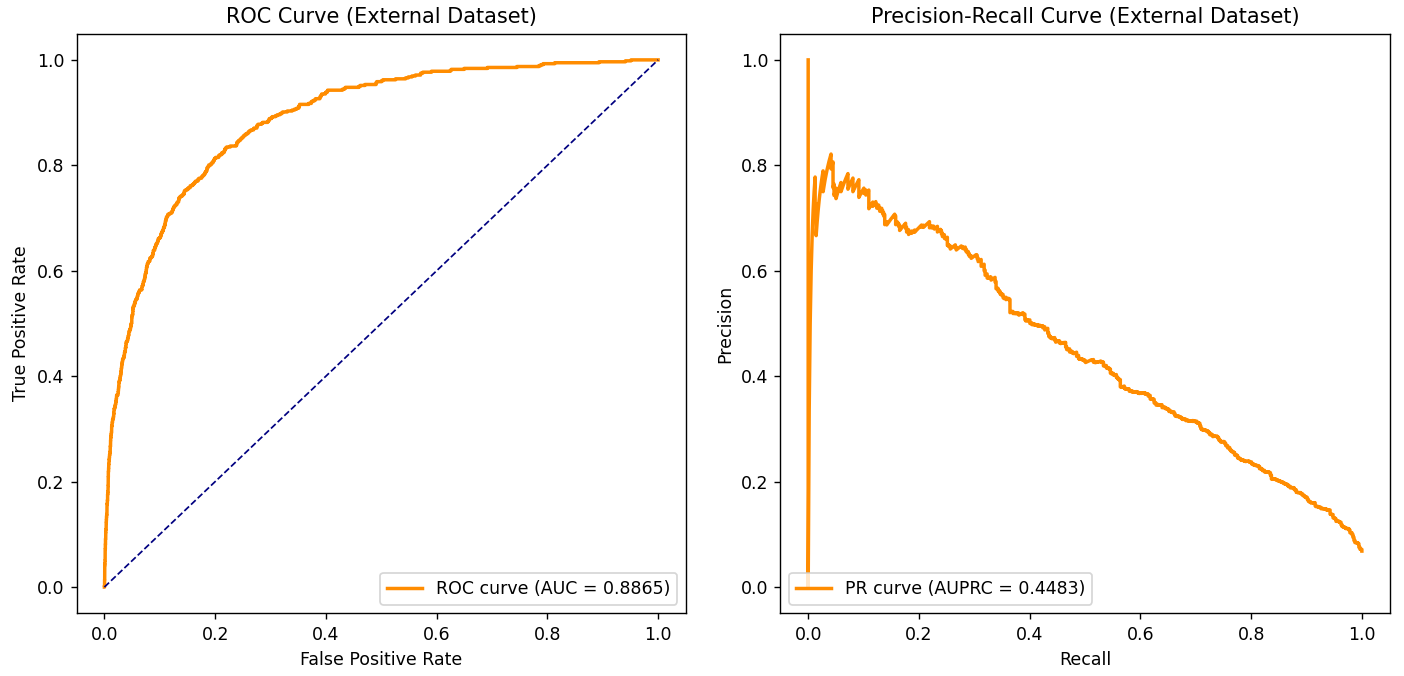


Supplementary Figure 6: ROC curve of XGBoost on external test set.


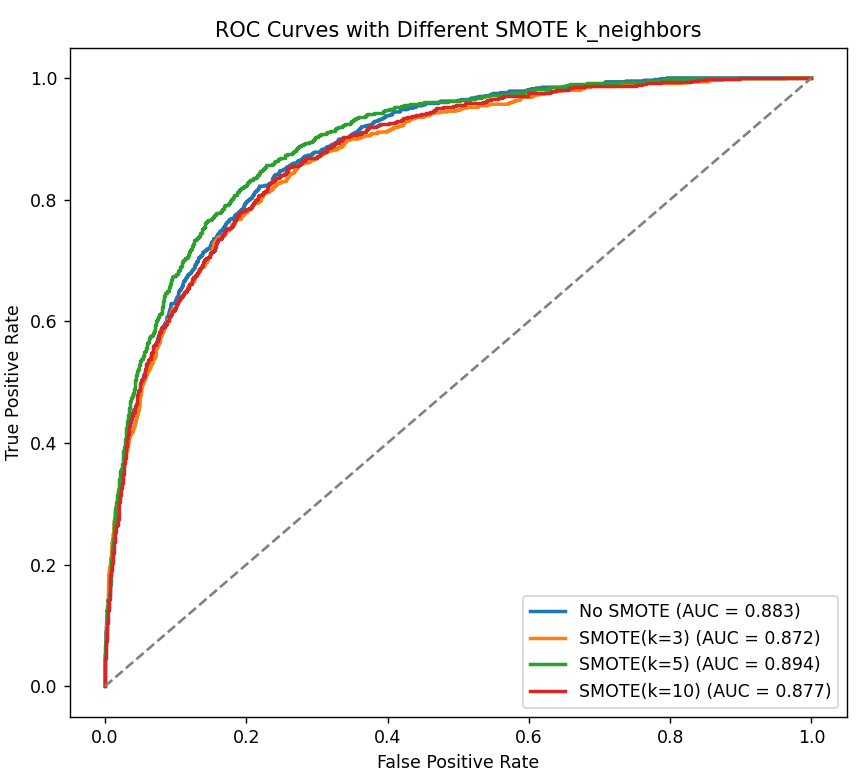


Supplementary Figure 7. ROC curves of XGBoost on the test set under different k values of SMOTE.


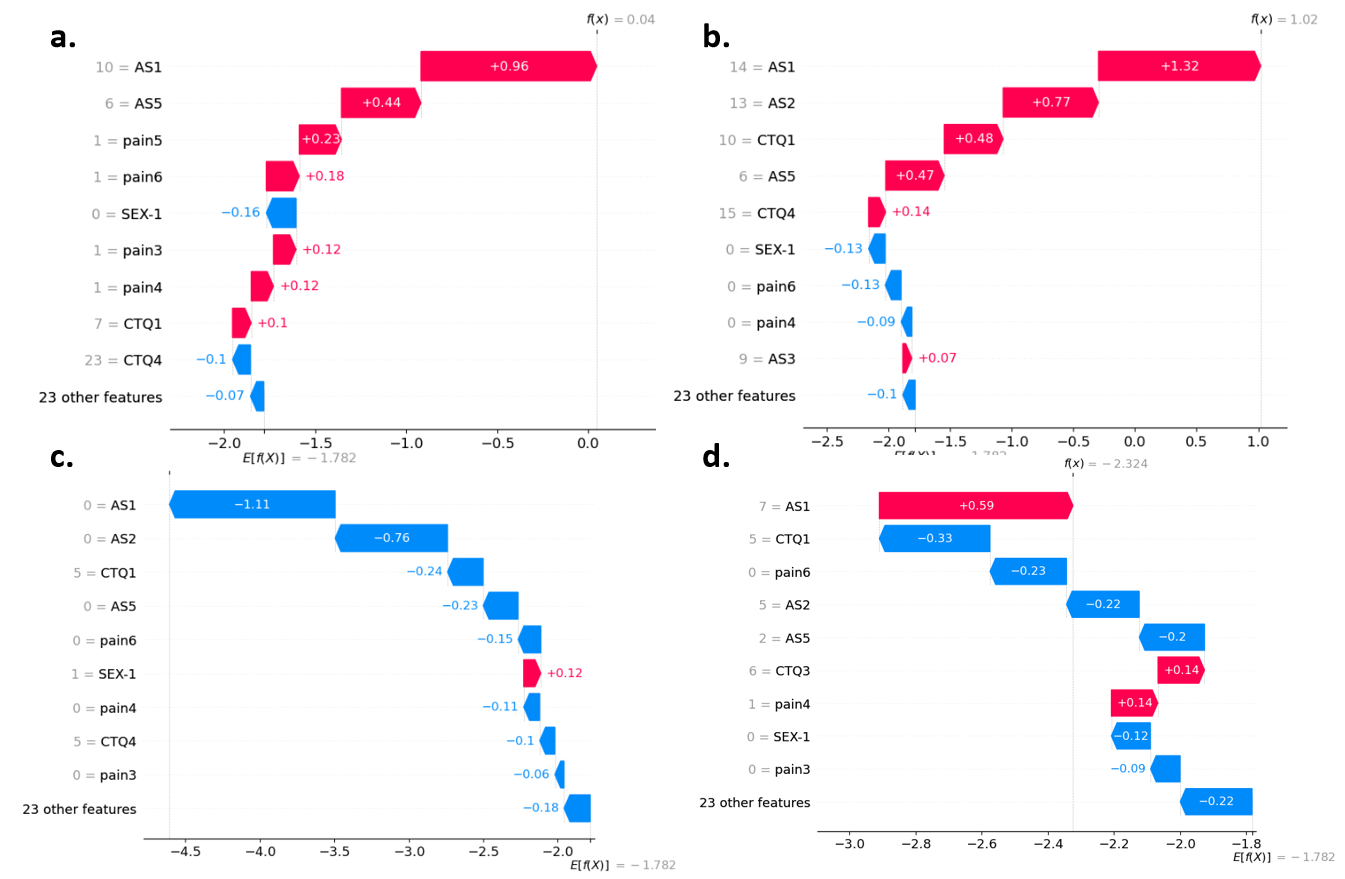


Supplementary Figure 8. Influence of features to specific samples. a. True Positive example. b. False Positive example. c. True Negative example. d. False Negative example.


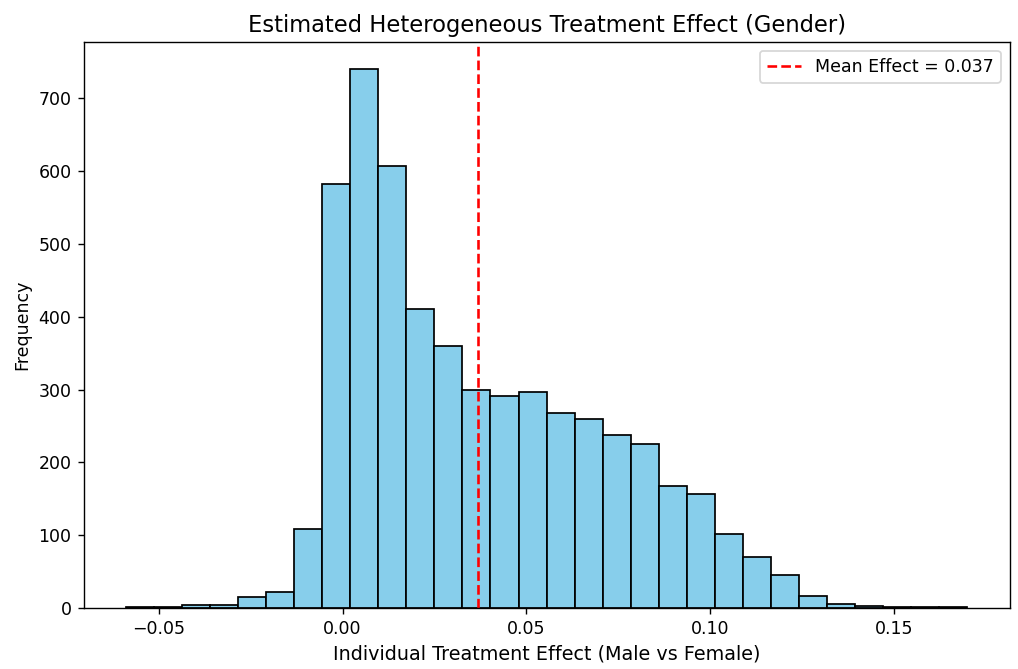


Supplementary Figure 9. Distribution of Individual Treatment Effects (ITEs) and Average Treatment Effect (ATE).


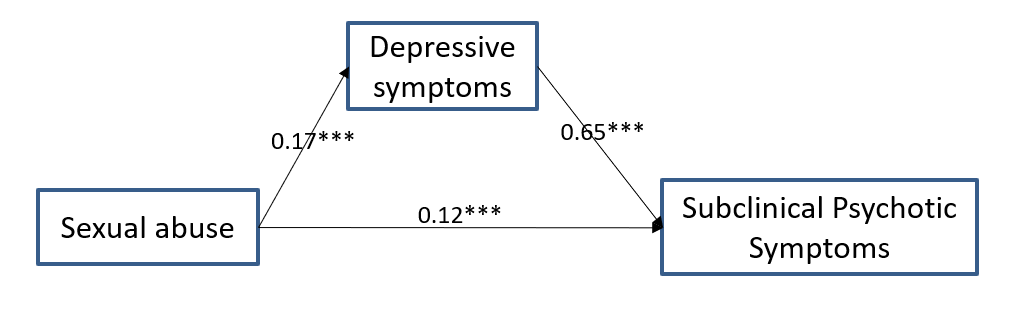


Supplementary Figure 10. Depressive symptoms mediated the relationship between childhood sexual abuse and SPS

**References**

1. Chen T, Guestrin C: **Xgboost: A scalable tree boosting system**. In: *Proceedings of the 22nd acm sigkdd international conference on knowledge discovery and data mining: 2016*; 2016: 785-794.

2. Breiman L: **Random forests**. *Machine learning* 2001, **45**:5-32.

3. Cortes C: **Support-Vector Networks**. *Machine Learning* 1995.

4. Chawla NV, Bowyer KW, Hall LO, Kegelmeyer WP: **SMOTE: synthetic minority over-sampling technique**. *Journal of artificial intelligence research* 2002, **16**:321-357.

5. Lundberg S: **A unified approach to interpreting model predictions**. *arXiv preprint arXiv:170507874* 2017.
